# Engaging community to co-design learning health systems: lessons from storytelling and Design Jam, a community case study from British Columbia, Canada

**DOI:** 10.3389/frhs.2025.1620659

**Published:** 2025-07-29

**Authors:** Margaret Chen-Mei Lin, Krisztina Vasarhelyi, Karen Lok Yi Wong, Haruka Furuichi, Jim Mann, Annette Berndt, Kayoung Lee, Lori Benning, Lillian Hung

**Affiliations:** ^1^ School of Nursing, University of British Columbia, Vancouver, BC, Canada; ^2^ IDEA Lab, University of British Columbia, Vancouver, BC, Canada; ^3^ Strategy Deployment, Vancouver Coastal Health, Vancouver, BC, Canada; ^4^ School of Nursing, University of Fukui, Fukui, Japan

**Keywords:** person centered health system, learning health systems, health services planning, patient and community engagement, co-creation, participatory action research, design thinking

## Abstract

Health and research systems produce vast amounts of data, yet only a fraction is used to improve healthcare delivery—especially for equity-deserving communities. In Canada, Learning Health Systems (LHS) are guided by the Quadruple Aim: improving population health, enhancing patient and provider experience, and reducing costs, with equity now recognized as a critical additional aim. As LHS evolve, advancing health equity has become a core driver, particularly in Canada. An equitable LHS prioritizes inclusion, accessibility, and co-creation, ensuring that historically marginalized communities are active partners in shaping healthcare solutions. Community engagement is foundational to LHS, where individuals, families, and communities collaborate with clinicians, researchers, and decision-makers to drive meaningful improvements. This community case study describes how a large health authority in British Columbia integrated design thinking and a participatory action research approach to co-develop a vision for a community-centered LHS. Fifty diverse partners participated, including individuals and families, clinicians, non-clinical health staff, health administrators, researchers, and students. The project team drew on a Canadian LHS framework, appreciative inquiry, and design thinking to guide engagement activities. Participants co-designed a vision for LHS, proposing actions across six key areas, including (1) Legal and Ethical, (2) Science and Research, (3) Data and Technology, (4) Policy, Process, and Resources, (5) Indigenous Leadership & Participation, (6) Social, Community, and Equity. Through the sessions, lived experiences helped surface barriers and community priorities. Storytelling and Design Jam methods were key tools for fostering meaningful engagement. We propose practical considerations (INSPIRE) that researchers and policymakers can apply to enhance participation, foster equity, and ensure that Learning Health Systems remain community-driven and responsive to diverse needs: Inclusion first, Nurture Trust, Show impact, Partner with lived experience experts, Institutionalize diverse engagement, Recognize ethical responsibilities, and Ensure sustainability. Future research should investigate how to overcome barriers to participation, embed participatory approaches, and consider design-thinking in health system transformation. By focusing on community engagement, this case study demonstrates how LHS can be co-developed as inclusive and equity-driven.

## 1 Introduction

In recent years, health systems worldwide have increasingly recognized the need to transform how healthcare knowledge is generated and applied to improve care outcomes and system efficiency (1, 2). Despite the vast amounts of data and evidence produced by health and research systems, only a fraction is effectively translated into practice, limiting its impact on healthcare improvement (3, 4). The Learning Health System (LHS) model was developed to bridge this gap by leveraging real-time data, collaborative research, and system feedback loops to facilitate evidence-based decision-making (5, 6). While LHS was not originally designed to address health disparities, it offers a valuable opportunity to create more inclusive and adaptive healthcare frameworks (3, 4).

Equity has emerged as a central driving force in LHS implementation, particularly in the Canadian context, where health disparities persist due to fragmented systems and a lack of meaningful community engagement (3, 7). The LHS Action Framework by Reid et al. (4) positions equity as the foundation of LHS, emphasizing co-design with patients, communities, and families. Without inclusive and adaptive frameworks, healthcare services often fail to address the unique needs of diverse populations, exacerbating disparities in access and outcomes. Integrating Equity, Diversity, and Inclusion (EDI) principles within LHS is essential to ensuring that all voices—particularly those from underserved groups—are actively involved in shaping healthcare systems (8, 9).

Participatory approaches have gained prominence as effective strategies for fostering collaboration, mutual learning, and shared power between researchers and community members. These methods ensure that healthcare interventions are contextually relevant and responsive to the needs of diverse populations (10, 11). Design Jam is a collaborative, fast-paced brainstorming activity rooted in design thinking (a person-centered approach popular in the designing industry that seeks to understand user needs and perspectives while fostering creative problem-solving opportunities) that well-aligned with participatory approaches (12, 13). Design Jam encourages divergent-thinking activities such as small group discussions to generate ideas rapidly, along with idea-converging activities such as big group debrief, to co-design solutions (13). It highly aligns with the project team's needs of a structured method to engage diverse partners—including patients, families, clinicians, researchers, and decision-makers—in co-developing innovative and equitable healthcare solutions such as a learning health system (14, 15). However, its application within LHS remains novel. This approach is particularly valuable for addressing the needs of historically marginalized and underrepresented communities, such as Indigenous populations, racialized groups, and low-income individuals, who often face systemic barriers—including historical injustices, cultural misunderstandings, socioeconomic disparities, and inadequate access to health services (16–18). The term “underrepresented” in this context refers to communities whose voices and needs are often excluded from mainstream healthcare policy discussions, leading to gaps in service provision and persistent health inequities. By fostering collaborative, culturally sensitive, and community-driven solutions, Design Jams can enhance the inclusivity and responsiveness of LHS models (10, 11, 19).

The purpose of this paper is to examine the role of community engagement in co-developing a Learning Health System (LHS) within a large health authority in British Columbia, Canada. Specifically, we analyze key elements that facilitated the participation of diverse stakeholders—including patients, families, clinicians, researchers, and decision-makers—in shaping an equity-driven LHS. This paper highlights the use of Design Jam as a participatory method to foster dialogue with diverse representation and drive meaningful health system improvements. By reflecting on this co-design process, we identify barriers, priorities, and actionable strategies to strengthen LHS implementation. Our findings contribute to the growing body of evidence supporting inclusive, community-centered, and equity-driven healthcare transformation (3, 19, 20).

## 2 Context

Vancouver Coastal Health (VCH) is a regional health authority in British Columbia, Canada that has recently begun integrating Learning Health System (LHS) principles into its healthcare approach. VCH serves a diverse population of 1.25 million people in urban, rural, and Indigenous communities, each with unique cultural and healthcare needs (21). By adopting the LHS model, an organization can create a health system that not only responds to immediate medical needs but also continuously adapts to improve long-term health outcomes for all communities it serves (22).

The project team leading this initiative consisted of 18 interdisciplinary members, including researchers, health administrators, engagement specialists and graduate students from VCH and partnering academic institutions. Their collective expertise ensured that the integration of LHS principles remained grounded in scientific and academic community priorities and evidence, to be further expanded with broader community priorities.

## 3 Key elements

The project team planned one storytelling session and one Design Jam. Storytelling is a powerful tool to surface lived experiences, fostering empathy and deeper understanding among diverse groups (23–26).The storytelling session aimed to strengthen the bond between participants prior to co-designing and provide insights for the Design Jam. The Design Jam then built on the stories shared to foster co-creation of a vision on LHS. This section describes how the two sessions occurred.

### 3.1 Storytelling session

To recruit participants and facilitate meaningful engagement, the project team collaborated with a specialized VCH partner engagement team to develop a dedicated webpage containing text and video information about the project, along with sign-up forms for participation. This webpage was shared with a diverse network of patients, families, and community partners who had previously expressed interest in research initiatives. Additionally, the project team identified and directly invited 83 health system members—including VCH clinicians, managers, directors, team leaders, data support staff, and researchers—via email.

A total of 26 participants from the community and the health system joined the online storytelling session via a video conferencing platform. At least one participant from each discipline was present. Table 1 provides an overview of the participant demographics.

**Table 1:** Storytelling session participant demographic.

Participant category	Number of participant (%)
Community Members	8 (31%)
Frontline Clinicians	3 (11%)
Leader/Manager	8 (31%)
Director/Senior Leader	4 (15%)
Data Support Staff	1 (4%)
Researcher	2 (8%)
**TOTAL**	**26** **(100%)**

#### 3.1.1 Facilitation and structure

Prior to the sessions, an email was sent to participants inviting them to reflect on both positive and negative personal experiences within VCH's community health services setting. In the email, a two-minute video explained the nature and purpose of the project and why participants' stories would greatly enhance future work. Team members including a VCH partner engagement specialist and a designer with facilitation experience developed a facilitation guide to support six facilitators who will support story sharing. The guide shared tips that can foster a safe environment for experience sharing, such as ensure everyone feels welcome and respected, recognize that stories may bring up uncomfortable memories and participant can choose to leave or seek support at any time, and make space for questions and dialogues.

The session began with the lead facilitator introducing the session's purpose, guiding participant introductions, and explaining that the shared stories will only be documented by notetakers in writing and used for Design Jam activity planning. The lead facilitator also informed every participant that a spare virtual room will be available with a team member in addition to the break-out rooms, if any participant chooses to leave a breakout room anytime and rest.

Following the introductions, the core project team introduced the concept of LHS and why storytelling from diverse partners is vital for co-creating a LHS. Participants were then assigned to pre-arranged virtual breakout rooms, ensuring representation from various disciplines. Each breakout room included three to five participants, one experienced facilitator from the VCH engagement team, and one volunteer graduate student serving as a notetaker. Facilitators posed two key questions to guide discussions:

1. What is working well in the VCH community?
2. What is not working well in the VCH community?

In addition to the two key questions, each facilitator also holds two presentation slides, each including six prompts that participants can refer to when sharing stories. For example, to encourage stories that address “What is working well…”, the prompts include “Tell us about a good healthcare experience, and what made it good,” and “In what ways has the collaboration between healthcare providers improved patient care and outcomes?” For “What is not working well…”, the prompts include “What are some factors that prevent you from providing or receiving good care?” and “What are some experiences in healthcare settings in which you felt uncomfortable and why?” The prompts used by the facilitators are shared in Table 2. After one hour of small group sharing, all participants reconvened in the main virtual room to share brief summaries of their discussions for 15 min. The session was not recorded to maintain confidentiality; instead, notetakers anonymized and documented participant insights on a secure drive. Each facilitator reviewed the notes from their respective breakout rooms, after which the project team conducted a collective review and analysis to synthesize key themes and better understand the current state of VCH's community, as shared by diverse participants.

**Table 2:** Prompts to facilitate story-sharing.

What is working well?	What is not working well?
• What support have you received upon care delivery that improved your experience significantly, and why? • What is your expectation on the scope of information and knowledge your healthcare providers have? • Tell us about a good healthcare experience, and what made it good. • In what ways has the collaboration between healthcare providers improved patient care and outcomes? • How has the integration of technology positively impacted the efficiency and effectiveness of healthcare delivery? • What are some great healthcare initiatives that involve the community?	• What information or resources do you need from your healthcare providers? • What are the challenges during the interaction with patients? • What are some factors that prevent you from providing or receiving good care? • What are barriers in navigating different healthcare resources available? • What are some experiences in healthcare settings in which you felt uncomfortable and why? • What are the barriers to accessing care in primary care settings? • Tell us about a frustrating healthcare experience, and what made it frustrating.

### 3.2 Design Jam workshop

The Design Jam took place in-person 6 months after the storytelling sessions. During the 6-month period, the project team met regularly to analyze insights from the storytelling session and planned for Design Jam activities. The primary goal was to create a welcoming and inclusive space where participants could collaboratively shape a potential vision for Learning Health Systems (LHS) at VCH and explore strategies for implementation.

To recruit participants, the project team sent email invitations to all individuals who participated in the storytelling session, as well as 98 interdisciplinary VCH staff members. A total of 26 individuals expressed interest, and 24 participants attended the in-person session. 7 of the 24 participants previously attended the storytelling session. Table 3 provides demographic details of the Design Jam participants.

**Table 3:** Design Jam participant demographic.

Participant category	Number of participant (%)
Community Member	5 (21%)
Frontline Clinicians	2 (8%)
Leader/Manager	6 (25%)
Director/Senior Leader	7 (29%)
Data Support Staff	1 (4%)
Researcher	3 (13%)
**TOTAL**	**24** **(100%)**

**Figure 1:**
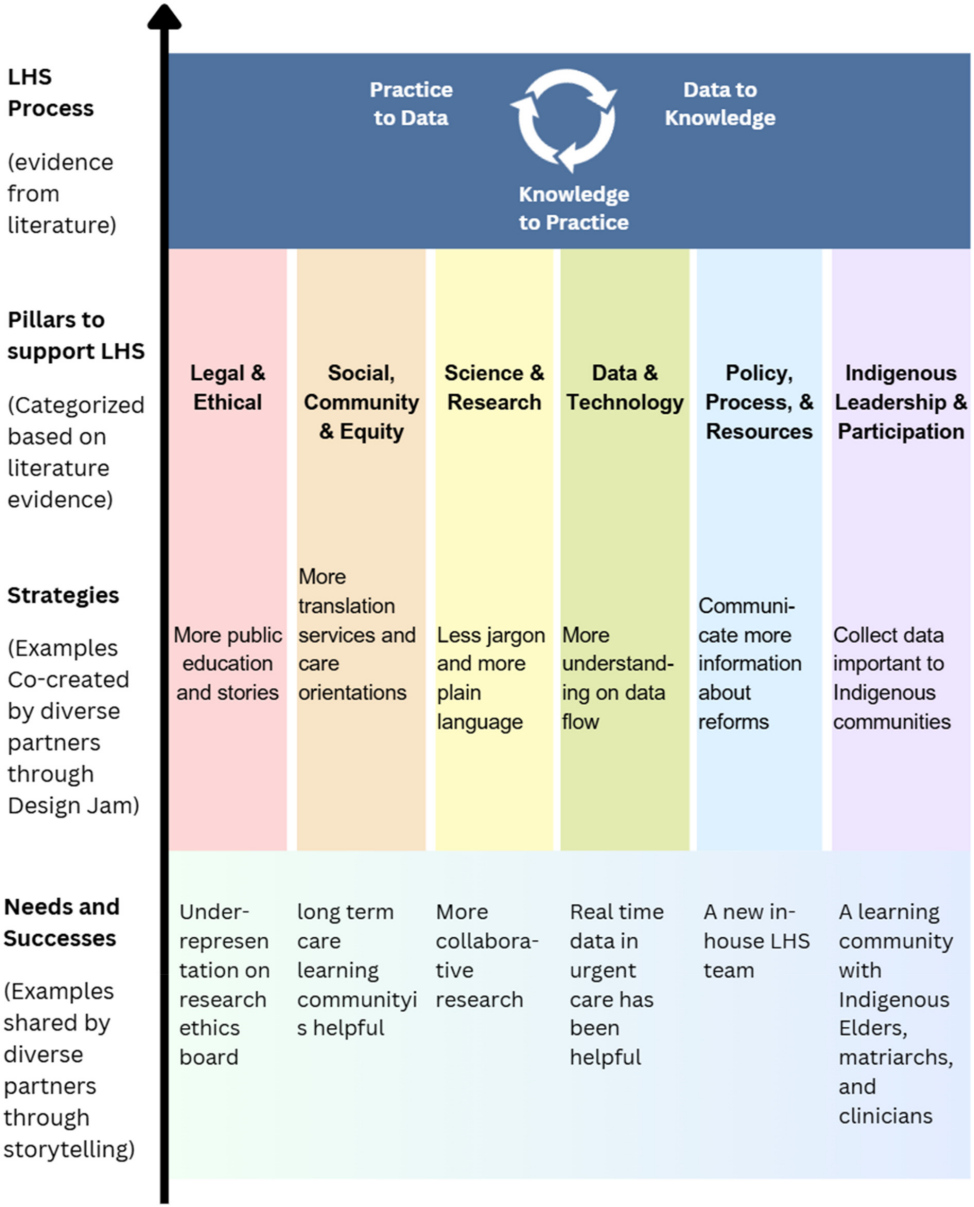
Structure where participant insights inform LHS. A diagram showing how participants insights (needs and success stories) from the storytelling session and co-created strategies from the design jam strengthen the six pillars identified by literature evidence, to be adopted into continuous learning cycles in the future. The six pillars are (1) Social, Community and Equity, (2) Legal and Ethical, (3) Science and Research, (4) Data and Technology, (5) Policy, Process and Resources, and (6) Indigenous Leadership and Participation. The diagram is informed and inspired by the framework from Menear et al. (3).

**Figure 2:**
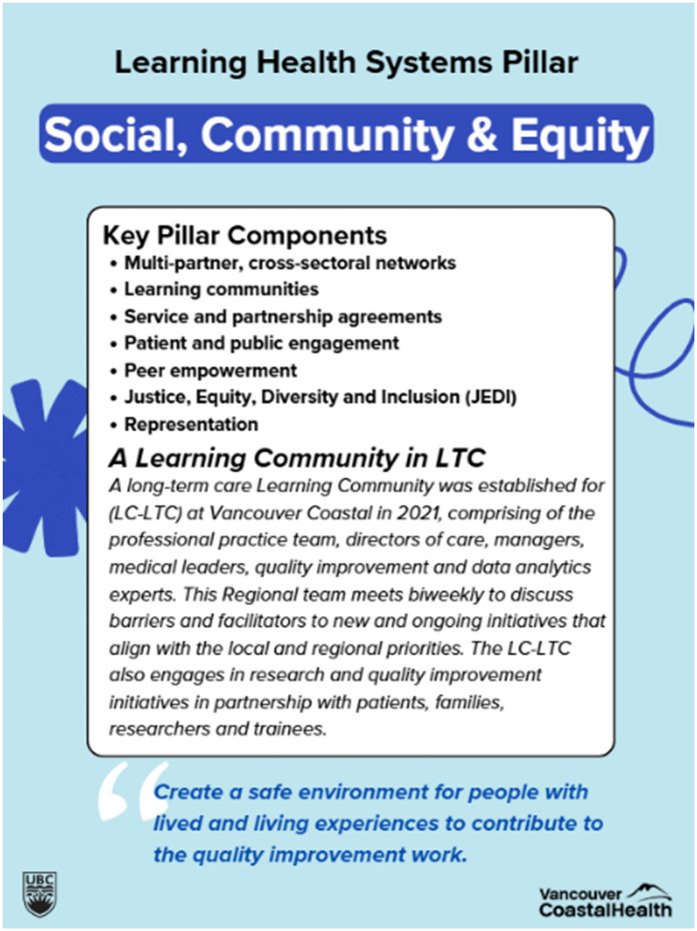
An example of a pillar poster used in the design Jam. The figure shows a blue poster that the project team used in the Design Jam. It includes key pillar components informed by literature, followed by one local success story that strengthens this pillar. The bottom of the poster shows a quote from the storytelling participant, stating a vision related to this pillar.

The project team synthesized themes from the stories shared in the previous storytelling session and integrated them into the workshop materials. For example, some stories reflected hopefulness on emerging initiatives such as virtual care and real-time data sharing and appreciation of compassionate health care teams, while many stories described frustrations on structural barriers to healthcare access, siloed care, and missing patient and family representations. These narratives were organized according to the LHS framework by Menear et al. (3), allowing the team to identify six tailored pillars to guide the development of an LHS for the VCH community: (1) Social, Community and Equity, (2) Legal and Ethical, (3) Science and Research, (4) Data and Technology, (5) Policy, Process and Resources, and (6) Indigenous Leadership and Participation. Each pillar was represented on a poster that outlined its key characteristics, informed by both existing literature and participant narratives from the storytelling sessions. Additionally, each poster featured a local story shared by participants in the storytelling session to illustrate practical applications of the LHS principles. Figure 1 shows how the themes from the storytelling session inform LHS, and Figure 2 provides an example of a workshop poster used during the session.

The three-hour Design Jam started with participant introductions and a brief summary of themes from the storytelling sessions. This was followed by a 15 min presentation by KV, who introduced the foundational concepts of Learning Health Systems (LHS), including frameworks and learning cycles from existing literature (3, 4, 27). Providing participants with a shared understanding of LHS helped establish a common purpose for the workshop.

The workshop setup included six discussion stations, each representing one of the six LHS pillars. Each station was equipped with tables, chairs, pillar posters, blank paper, sticky notes, markers, printed instructions, and snacks. A facilitator and a notetaker—either researchers or graduate students with experience in leading discussion groups—were assigned to each station.

#### 3.2.1 Participant engagement and discussion structure

Before the workshop, the pillar posters were shared via email with all registered participants, who were asked to select two pillars they were most interested in discussing. During the session, participants rotated through their two chosen small-group discussions, spending 30 min at each station.

Each small group addressed two guiding questions:

1. How can we strengthen this pillar to support an LHS at Vancouver Community?
2. How can we break down silos and align the pillars for better integration?

Participants were encouraged to contribute ideas by writing on sticky notes and posting them on the pillar boards or sharing them verbally while notetakers documented key points. After completing the two rounds of small-group discussions, all participants had a lunch break where they were encouraged to browse the pillar boards from different groups. Participants then reconvened for a 30 min plenary session, where facilitators and participants shared key takeaways and reflections.

**Figure 3:**
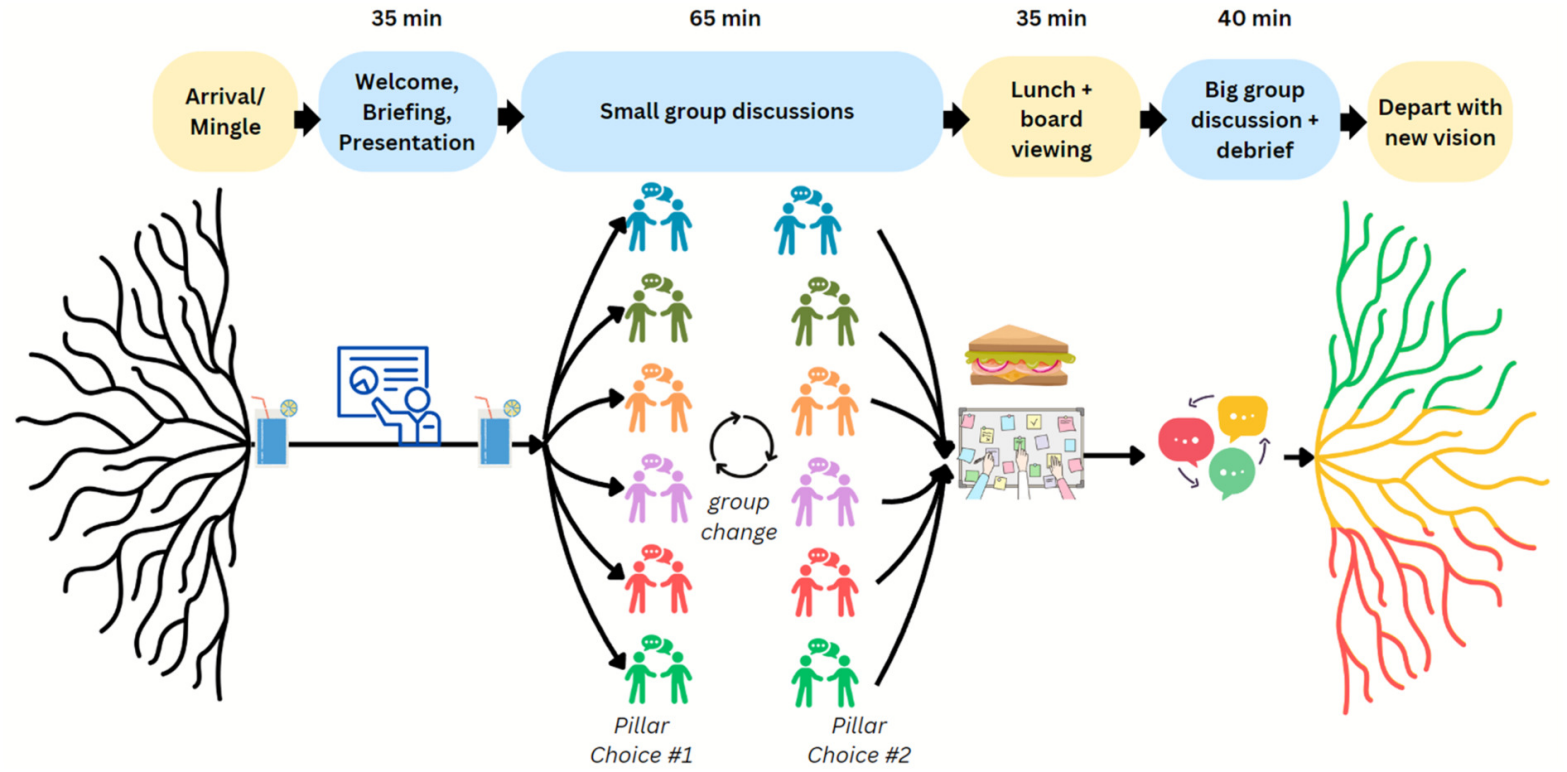
Design Jam process. A flowchart with five stages of the Design Jam: Arrival/Mingle, Welcome and presentations, Small group discussions (with a group change in the middle), Lunch and board viewing, and Big group discussion and debrief, each represented with simple icons and arrows connecting them.

At the end of the session, the project team collected all sticky notes and written inputs, which were transcribed into an electronic document for further analysis. Figure 3 illustrates the Design Jam process in a visual format.

## 4 Discussion

Our experience in co-developing a Learning Health System (LHS) through storytelling sessions and a Design Jam workshop has provided several key insights that may inform future community collaborations aimed at building a more inclusive and equity-driven LHS. The participatory approach facilitated meaningful engagement, interdisciplinary collaboration, and the co-creation of actionable solutions, reinforcing the importance of sustained community involvement in health system transformation.

### 4.1 Inclusive participation

A key lesson from our case study was the importance of engaging a broad and diverse group of participants, rather than limiting involvement to a small, select group. This was particularly crucial in ensuring that community partners with lived experience—who are often overlooked in policy and service development—had a meaningful role in shaping the LHS vision. By prioritizing diverse participation, we captured a wide range of perspectives, enriching our collective understanding of how an LHS can be structured to address systemic inequities. This aligns with existing literature, which highlights the persistent underrepresentation of diverse voices, particularly those with lived experience, in healthcare policy development (16–18). Our case study demonstrated that meaningful inclusion of these voices is achievable through structured engagement methods such as storytelling and Design Jams.

The Design Jam discussions revealed both opportunities and challenges in LHS implementation. While participants valued the six tailored LHS pillars, they also identified areas where fragmented health system processes create barriers to integration. Discussions emphasized that silos between clinical care, research, and policy must be broken down, reinforcing the need for better alignment between governance structures, frontline healthcare providers, and community stakeholders. These insights will inform the planning and direction of future LHS discussions in the health authority and beyond.

### 4.2 Addressing barriers to engagement

Another critical takeaway was recognizing and accommodating the varied needs of participants, particularly those from traditionally underrepresented backgrounds, to facilitate their full and meaningful engagement. A one-size-fits-all approach does not work in diverse settings, and flexibility in participation formats is necessary to ensure accessibility.

To address participation barriers, a hybrid model was implemented, allowing participants with mobility challenges to join virtually while offering an in-person option for those who preferred face-to-face discussions. Financial and transportation support was provided to participants facing economic or logistical barriers. This included travel subsidies, special transportation arrangements, and coordination with care partners to facilitate participation. Multiple modes of engagement (e.g., verbal discussions, written contributions via sticky notes, and facilitated breakout rooms) ensured that all participants could contribute in ways that suited their comfort level. These efforts built trust and credibility among participants, demonstrating that their contributions were valued and that the project team was committed to fostering an inclusive and equitable discussion.

### 4.3 Sustaining impact and participant expectations

A final key lesson was the importance of demonstrating how participant contributions would have a lasting impact on the ongoing development of an LHS. Throughout the collaboration, participants consistently asked how their input would be used beyond the sessions. In response, the project team provided updates on future plans, integrated their feedback into the LHS framework, and invited ongoing engagement. This transparency was highly valued and reinforced participants' sense of agency and ownership over the LHS vision.

Similar findings have been reported in previous collaborative research projects involving community members and individuals with lived experience (10, 11). The repeated inquiries from participants about the long-term impact of their contributions highlighted the necessity of consistent feedback loops and ongoing engagement strategies. Ensuring that community contributions continue to inform LHS development is critical for building trust and maintaining participant investment in the process.

### 4.4 Ethical considerations and broader implications

Beyond these practical insights, our case study contributes to the growing body of research on participatory health system transformation. Previous studies have shown that excluding underrepresented voices in policy and service development leads to policies that fail to meet their needs, exacerbating health disparities (16–18). LHS offers a unique opportunity to embed inclusive and equity-focused decision-making processes, fostering shared ownership, mutual learning, and sustained collaboration (3, 4). Our study provides a concrete example of how participatory approaches—such as Design Jams and storytelling—can be operationalized in LHS development. By prioritizing inclusivity, accommodating diverse needs, and ensuring long-term engagement, this case study demonstrates how LHS can be co-created with and for communities, ultimately leading to more adaptive, responsive, and equitable healthcare systems.

Embedding Design Jams and storytelling as recurring engagement tools within Learning Health System planning can help sustain momentum and ensure that equity remains central to system transformation. Further research should explore the longitudinal impacts of participatory engagement, particularly how co-designed solutions evolve over time and adapt to changing healthcare needs. Additionally, identifying strategies for institutionalizing participatory decision-making within health authorities is essential to prevent engagement efforts from becoming one-time initiatives rather than sustained practices, and ensure system improvements align with individuals' needs and expectations. Finally, developing metrics to evaluate the effectiveness of inclusive LHS models will be crucial in assessing their impact on healthcare accessibility, equity, and system efficiency, ensuring that participatory approaches translate into tangible improvements in health outcomes.

### 4.5 Lesson learned and practical considerations: INSPIRE

To ensure inclusive and meaningful engagement in Learning Health Systems (LHS), the following INSPIRE considerations highlight key lessons learned and practical recommendations from our study:

I—Inclusion First
  People have diverse needs, requiring intentional inclusivity in engagement strategies.
  Offer multiple participation options (e.g., Zoom for those with mobility challenges, in-person for those who struggle with virtual settings).
  Provide accommodations such as HandyDart transportation or caregiver support to remove participation barriers.
N—Nurture Trust
  Establish clear communication and build relationships with participants to create a safe and welcoming space.
  Acknowledge past exclusion and demonstrate genuine commitment to incorporating diverse perspectives into decision-making.
S—Show Impact
  Clearly demonstrate how participant contributions shape the LHS and broader healthcare policies.
  Provide updates and follow-ups to show how feedback translates into real-world change.
  Reinforce why their voices matter, ensuring that engagement is not perceived as tokenistic.
P—Partner with Lived Experience Experts
  Recognize community members as experts in their own experiences.
  Collaborate with people with lived experience in co-designing solutions, rather than just consulting them after decisions have been made.
  Validate insights from lived experience as equally valuable to academic and professional expertise.
I—Institutionalize Diverse Engagement
  Move beyond small, select groups—engage broader, more diverse communities to capture a fuller range of perspectives.
  Ensure that underrepresented voices are consistently integrated into LHS policy and service development.
R—Recognize Ethical Responsibilities
  Ethical engagement requires fair representation, accessibility, and cultural safety.
  Address historical injustices and power imbalances that may influence participation dynamics.
  Follow principles from existing frameworks, such as the Lived Experience in Policymaking Guide (28).
E—Ensure Sustainability
  Long-term engagement strategies are necessary to maintain momentum and drive meaningful change.
  Embed participatory approaches into institutional policies to prevent short-term, one-off initiatives.
  Develop structured mechanisms to sustain community involvement in decision-making.

## 5 Conclusion

Our findings contribute to the literature by emphasizing the necessity of engaging diverse groups, rather than limiting input to a small, select few. Community partners with lived experience must be integrated meaningfully into LHS development to impact services and policies effectively. Trust, diverse knowledge, sustainability, and ethical engagement are key principles that must underpin participatory health system transformation. Engagement and co-design methods such as Design Jams and Storytelling sessions can be helpful in fostering LHS co-creation. By applying INSPIRE considerations, researchers and policymakers can enhance participation, foster equity, and ensure that Learning Health Systems remain community-driven and responsive to diverse needs.

## Data Availability

The original contributions presented in the study are included in the article/Supplementary Material, further inquiries can be directed to the corresponding author.
